# Methods for teaching evidence-based practice: a scoping review

**DOI:** 10.1186/s12909-019-1681-0

**Published:** 2019-07-11

**Authors:** Camilla Marie Larsen, Anne Seneca Terkelsen, Anne-Marie Fiala Carlsen, Hanne Kaae Kristensen

**Affiliations:** 1 Health Sciences Research Centre, UCL University College, Niels Bohrs Allé 1, 5230 Odense M, Denmark; 2 Department of Physiotherapy, UCL University College, Niels Bohrs Allé 1, 5230 Odense M, Denmark; 3 Research Service, UCL Library, UCL University College, Niels Bohrs Allé 1, 5230 Odense M, Denmark; 4 Research Unit for Musculoskeletal Function and Physiotherapy, Department of Sports Science and Clinical Biomechanics, University of Southern Denmark, Campusvej 55, 5230 Odense M, Denmark; 5 Department of Clinical Research, University of Southern Denmark, Winsløwparken 19, 5000 Odense, Denmark

**Keywords:** Teaching methods, Undergraduate healthcare students, Evidence-based practice, The Sicily statement

## Abstract

**Background:**

This scoping review aims to gather and map inspiration, ideas and recommendations for teaching evidence-based practice across Professional Bachelor Degree healthcare programmes by mapping literature describing evidence-based practice teaching methods for undergraduate healthcare students including the steps suggested by the Sicily Statement.

**Methods:**

A computer-assisted literature search using PubMed, Cinahl, PsycINFO, and OpenGrey covering health, education and grey literature was performed. Literature published before 2010 was excluded. Students should be attending either a Professional Bachelor’s degree or a Bachelor’s degree programme. Full-text articles were screened by pairs of reviewers and data extracted regarding: study characteristics and key methods of teaching evidence-based practice. Study characteristics were described narratively. Thematic analysis identified key methods for teaching evidence-based practice, while full-text revisions identified the use of the Sicily Statement’s five steps and context.

**Results:**

The database search identified 2220 records. One hundred ninety-two records were eligible for full-text assessment and 81 studies were included. Studies were conducted from 2010 to 2018. Approximately half of the studies were undertaken in the USA. Study designs were primarily qualitative and participants mainly nursing students. Seven key methods for teaching evidence-based practice were identified. Research courses and workshops, Collaboration with clinical practice and IT technology were the key methods most frequently identified. Journal clubs and Embedded librarians were referred to the least. The majority of the methods included 2–4 of the Sicily Statement’s five steps, while few methods referred to all five steps.

**Conclusions:**

This scoping review has provided an extensive overview of literature describing methods for teaching EBP regarding undergraduate healthcare students. The two key methods Research courses and workshops and Collaboration with clinical practice are advantageous methods for teaching undergraduate healthcare students evidence-based practice; incorporating many of the Sicily Statement’s five steps. Unlike the Research courses and workshop methods, the last step of evaluation is carried out partly or entirely in a clinical context. Journal clubs and Embedded librarians should be further investigated as methods to reinforce existing methods of teaching. Future research should focus on methods for teaching EBP that incorporate as many of the five steps of teaching and conducting EBP as possible.

## Background

Dizon et al. state that healthcare can be inefficient, ineffective and/or dangerous when it is not based on current best evidence [1, 2]. Therefore, to ensure the quality of healthcare, it is important to implement evidence-based practice (EBP) in all health professional curricula, so that future health professionals learn the fundamentals of research and the application of evidence in practice [2].

Several definitions of EBP have been suggested in recent years. The scientific evidence was initially developed within medicine, but as many health professionals have embraced an evidence-based way of practice the Sicily Statement [3] suggested that the original term “evidence-based medicine” should be expanded to “evidence-based practice” in order to reflect a common approach to EBP across all health professions.

The Sicily Statement gives a clear definition of EBP together with a description of the minimum level of educational requirements and skills required to practice in an evidence-based manner. This makes the underlying processes of EBP more transparent and distinguishes between the process and outcome of EBP [3].

In order to fulfil the minimum requirements of teaching and conducting EBP, the Sicily Statement puts forward a five-step model: (I) asking a clinical question; (II) collecting the most relevant evidence; (III) critically appraising the evidence; (IV) integrating the evidence with one’s clinical expertise, patient preferences and values to make a practice decision; and (V) evaluating the change or outcome [4].

Internationally, EBP skills are essential requirements in clinical practice among both medical doctors as well as among other health professionals. Healthcare students are mainly taught the first three steps of the Sicily Statement’s five-step model. The last two steps are rarely taught, and students and graduates thus lack competencies in applying their knowledge in the clinical setting during or after graduation [5, 6].

In terms of healthcare policy and ambitions in Denmark, it was decided in 2015 that Professional Bachelor Degree healthcare students were to contribute to the development of an evidence-based way of working, a faster implementation of new knowledge in practice, and to the development of greater patient involvement and patient safety in the Danish healthcare system [7]. The Professional Bachelor’s degree is awarded after 180–270 ECTS and includes a period of work placement of at least 30 ECTS. The programmes are applied programmes. They are development-based and combine theoretical studies with a practical approach. Examples of professional bachelor degree holders are nurses. The Danish title is Professionsbachelor and the English title is Bachelor [8]. In Denmark the University College institutions solely provide professional bachelor degree educations. Master degrees are awarded at the Universities.

Based on the Sicily Statement students should be able to reflect, ask questions, gather knowledge, critically appraise, apply and evaluate various kinds of knowledge at the end of their course. The aim is that all Professional Bachelor Degree healthcare students across disciplines of nursing, physiotherapy, occupational therapy, radiography, and biomedical laboratory science develop common EBP qualifications in order to contribute towards the development of evidence-based healthcare [9]. In order to ensure shared prerequisites and mutual understanding of the EBP concepts before entering theoretical or clinical inter-professional education, further knowledge about how to teach EBP across disciplines is required [9]. By teaching the fundamental principles of EBP, students will develop their EBP skills and ability to put them into practice in their studies and as future graduates.

Previously, some systematic reviews were conducted summarising various educational interventions or strategies for teaching EBP to undergraduate healthcare students [2, 10–12].

In a review from 2014, Young and colleagues stated that multifaceted interventions integrated into clinical practice contributed to the greatest improvements in EBP knowledge, skills, and attitudes [2]. In line with this, Kyriakoulis et al. suggested that a combination of interventions, such as lectures, tutorials, workshops, conferences, journal clubs, and online sessions was best suited for teaching EBP to undergraduate healthcare students [10]. However, the majority of the articles in both reviews synthesized information from interventions or strategies aimed at medical students at various educational levels. Only a few articles elicited information about educational interventions and strategies aimed at undergraduate healthcare students in the disciplines of nursing, physiotherapy, occupational therapy, radiography, and/or biomedical laboratory science. However, two recent reviews have specifically addressed EBP teaching for undergraduate nursing students [11, 12]. A systematic review investigated the effectiveness of specific educational methods and found an effect on student knowledge, attitudes, and skills but could not draw a conclusion as to the advisability of one of the methods [11]. A literature review sought to identify knowledge experiences on teaching strategies from qualitative studies in nursing EBP education to enhance knowledge and skills and points to a limited focus on the use of EBP teaching strategies. Additionally, the study points to the need for more qualitative research investigating interactive and clinically integrated teaching strategies. Despite both reviews being well-informing, a broad scope when mapping updated EBP teaching methods and strategies across healthcare bachelor educations will further qualify future interdisciplinary practices [11, 12].

In order to implement the most effective ways of teaching EBP across healthcare undergraduate students, an investigation of existing literature on the subject needs to be undertaken. For identifying, mapping and discussing key characteristics in the literature a scoping review is the better choice [13].

## Aim, objectives and review question

The aim of this scoping review is to gather and map inspiration, ideas, and recommendations for teachers implementing EBP across Professional Bachelor Degree healthcare programmes by mapping existing literature describing EBP teaching methods, including the five steps of EBP suggested by the Sicily Statement, [3] regarding undergraduate healthcare students.

The primary question of the scoping review is: “Which EBP teaching methods, including The Sicily Statement’s steps of teaching and conducting EBP, have been reported in the literature with respect to undergraduate healthcare students in classrooms and clinical practice?”

### Definitions

Classroom is defined as a room where classes are taught in a school, college or university [14].

Clinical practice refers to the agreed-upon and customary means of delivering healthcare by doctors, nurses and other health professionals [15].

## Methods

To ensure a systematic methodology, The Joanna Briggs Institute Reviewers’ Manual - Methodology for JBI Scoping Reviews has been used throughout the scoping review process [16, 17].

### Inclusion criteria

#### Participants

Literature which included undergraduate healthcare students in the disciplines of nursing, physiotherapy, occupational therapy, radiography, and biomedical laboratory science was selected to ensure applicability and relevance to similar scientific disciplines at other institutions of higher education. The undergraduate students should be attending either a Professional Bachelor’s degree or a Bachelor’s degree programme.

#### Concept

Methods for teaching EBP including The Sicily Statement’s steps of teaching and conducting EBP was the main concept to be investigated in the review. That is; literature describing either recommendations of EBP teaching methods, evaluations of EBP teaching methods, teacher and/or student perceptions of EBP teaching and learning methods, or qualifications obtained when learning the principles of EBP.

#### Context

Literature describing methods for teaching EBP conducted in a classroom setting, in clinical practice as part of the education, or in a combination of classroom and clinical practice was included in the review.

### Exclusion criteria

In the period up to 2010, the Bachelor Degree healthcare educations began to conform to European requirements regarding evidence-informed and evidence-based education [18].

A maximum time frame (2010–2018) was applied, determined by the amount of available literature/research studies and requirements of updated teaching strategies [19, 20]. Therefore, literature published before 2010 was excluded.

Literature including undergraduate students in other health disciplines such as medicine or dentistry was not reviewed as the structure of their education is based on another paradigm. Nor was literature including participants such as graduates, RN-to-BSN students, and trained health personnel accepted for inclusion as they were considered as postgraduates, not comparable to undergraduate students. With the primary aim of gathering ideas and inspiration for teaching EBP, literature that focused on issues other than methods for teaching EBP was excluded, as well as literature in languages other than English, Danish, Norwegian, or Swedish.

### Search strategy

To identify literature relevant to our research question, the databases MEDLINE via PubMed, CINAHL Complete, and PsycINFO (both via EBSCO) were systematically searched. These databases cover both health and education and are available to the primary local target audience of this scoping review. Because of time limitations only the multidisciplinary European database, OpenGrey, was searched in the attempt to find unpublished literature. The searches were conducted May 9th, 2018.

As recommended in The Joanna Briggs Institute Reviewers’ Manual [16, 17], the search was conducted in three steps in collaboration with a research librarian.

- Step 1: The databases PubMed, covering the field of biomedicine and CINAHL, covering nursing and allied health literature were searched using the keywords: ‘teaching methods’, ‘teaching’, ‘learning methods’, ‘learning’, ‘teaching strategies’, ‘learning strategies’, ‘undergraduate’, ‘undergraduate education’, ‘student’, ‘biomedical laboratory scientist’, ‘medical laboratory scientist’, ‘medical laboratory technologist’, ‘medical laboratory technologists’, ‘radiographer’, ‘occupational therapist’, ‘physiotherapist’, ‘nurse’, and ‘evidence-based practice’.
- Step 2: Through an analysis of text words in titles and abstracts of the studies found in PubMed and Cinahl, new keywords, which would improve the search, were identified. These were ‘allied health’, health students’, and ‘nursing’. All identified keywords were then included in the search as a systematic block search in PubMed, Cinahl, and PsycINFO, covering literature in the behavioural and social sciences, and OpenGrey, covering grey literature in Europe. Table 1 provides a list of the specific search queries used in all databases.
- Step 3: The reference lists of identified studies were searched for additional studies.

**Table 1:** Specific search queries, all databases

Database	Search queries
PubMed	(((((teaching OR learning))) AND (undergraduate OR student OR allied health OR health students)) AND ((biomedical laboratory scientist OR medical laboratory scientist OR medical laboratory technologist OR medical laboratory technologists OR radiographer OR occupational therapist OR physiotherapist OR nurse OR nursing))) AND evidence-based practice
Cinahl Complete	(teaching OR learning) AND (undergraduate OR student OR allied health) AND (biomedical laboratory scientist OR medical laboratory scientist OR medical laboratory technologist OR medical laboratory technologists OR radiographer OR occupational therapist OR physiotherapist OR nurse OR nursing) AND evidence-based practice
PsycInfo via EBSCO	(teaching OR learning) AND (undergraduate OR student OR allied health) AND (biomedical laboratory scientist OR medical laboratory scientist OR medical laboratory technologist OR medical laboratory technologists OR radiographer OR occupational therapist OR physiotherapist OR nurse OR nursing) AND evidence-based practice
Open Grey	(“Evidence based practice” OR EBP OR Evidence-based practice OR Evidence based practice) AND (teaching OR education OR learning) AND (undergraduate OR student OR students)

### Study selection

All search results from the databases were imported to the web-based bibliographic management software, RefWorks 2017 by ProQuest. After exclusion of duplicates and records before 01.01.2010, two reviewers independently screened titles and abstracts of the remaining articles for relevance in relation to the research question and the inclusion and exclusion criteria. Afterwards, all full-text articles were further checked for relevance by two independent reviewers. Any inconsistencies between the two reviewers regarding study selection for final inclusion were resolved by discussion with a third reviewer.

### Data collection

Data from the included articles were extracted using two data extraction tools as recommended in The Joanna Briggs Institute Reviewers’ Manual [16, 17]. The first data extraction tool comprised study characteristics, while the other data extraction tool comprised methods for teaching EBP.

Prior to the process of extracting data from the included articles, a pilot test using the data extraction tools was conducted by one reviewer assessing nine articles. To ensure agreement between reviewers, a second reviewer checked the same articles. Any disagreements about the content or use of the data extraction tools were discussed and resolved.

One reviewer then extracted relevant data from all included articles to the data extraction tools. Two other reviewers split the same articles among them and extracted data using the same data extraction tools. As a final step, the first reviewer went through all extracted data from all of the included articles with each of the other reviewers to ensure comparability and completeness in the final data extraction tools.

### Synthesis and analysis of results

The data extraction tools formed the basis of the final presentation of the results in two tables consisting of “Study characteristics” and “Key methods for teaching EBP, the Sicily Statement’s five steps of teaching and conducting EBP and context”. Study characteristics included author, year of publication, title, journal, country of origin, study design, study participants, methods for teaching EBP, and main study findings. The key methods for teaching EBP were identified through a thematic analysis. All full text articles were read and every teaching method found was listed. Through a revision of all teaching methods listed, seven themes were found that described the most prominent teaching methods, which were named “Key methods for teaching EBP”. All methods were then divided into one of the key methods for teaching EBP. In some articles, more than one teaching method was described. In that case, the teaching method most frequently described was selected and categorised under the relevant key method. Through full-text revision the Sicily Statement’s steps of teaching and conducting EBP and the context (classroom, clinical practice or a combination of both) in which the teaching took place was found. To further clarify the content of the two tables all results listed were described narratively. All tabulated data, except for the key methods for teaching EBP identified in Table 3, have been cited directly from the articles.

## Results

### Literature search

**Fig. 1:**
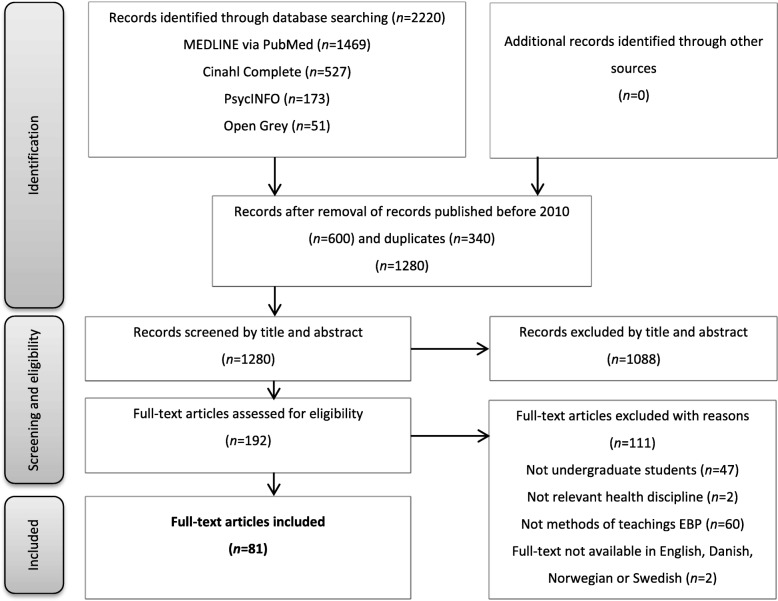
Modified PRISMA flow diagram illustrating the scoping review selection process

The database search returned 2220 records: PubMed (*n* = 1469), Cinahl (*n* = 527), PsycINFO (*n* = 173), and OpenGrey (*n* = 51) (Fig. 1). Records published before 01.01.2010 and duplicates were removed, which left 1280 records to be screened by title and abstract. Based on relevance, 1088 records were excluded and 192 records were found eligible for full-text assessment. In accordance with the inclusion and exclusion criteria, 111 articles were excluded. The excluded articles concerned study participants other than undergraduate healthcare students (graduates, RN-to-BSN students, trained health personnel), study participants from other healthcare disciplines (medicine, dentistry, midwifery), issues other than methods for teaching EBP (simulation teaching, community health nursing, EBP beliefs, etc.), and full-text articles not available in English, Danish, Norwegian, and Swedish (French, Chinese). In agreement with the other reviewers, 81 studies were finally included in the scoping review.

### Study characteristics

Study characteristics are presented in Table 2. All studies were spread across the years 2010–2018. Almost half of the studies (*n* = 40) were conducted in the USA, followed by Canada (*n* = 8), Norway (*n* = 7), Australia (*n* = 6), England (*n* = 6), Sweden (*n* = 3), China (*n* = 2), Finland (*n* = 2), Spain (*n* = 2), Greece (*n* = 1), Iran (*n* = 1), Lebanon (*n* = 1), Scotland (*n* = 1), and Taiwan (*n* = 1). The study designs were primarily qualitative (*n* = 55), while 23 of the studies were quantitative, and three of the studies used a mixed method. The majority of the participants were nursing students (*n* = 72), followed by a combination of nursing students and students from other healthcare disciplines (*n* = 5), nursing and physiotherapy students (*n* = 1), physiotherapy students and students from other healthcare disciplines (*n* = 1), occupational and physiotherapy students (*n* = 1), and physiotherapy students only (*n* = 1).

**Table 2:** Study characteristics (*N* = 81)

First author	Year	Title	Journal	Country	Design	Participants	Methods for teaching EBP	Main study findings
Aglen [21]	2016	Pedagogical strategies to teach bachelor students EBP: A systematic review	Nurse Education Today	Norway	Qualitative	Nursing students	Theories of discretion, knowledge transfer and cognitive maturity development	Nursing students struggle to see the relevance of evidence for nursing practice. Before being introduced to information literacy and research topics, students need insight into knowledge transfer and their own epistemic assumptions. Knowledge transfer related to clinical problems should be the learning situations prioritised when teaching EBP at bachelor level.
André [22]	2016	Embedding evidence-based practice among nursing undergraduates: Results from a pilot study	Nurse Education in Practice	Norway	Qualitative	Nursing students	Information about voluntary participation in two different clinical research projects, education programme related to EBP, participation in clinical research projects, instructions and education in analysing and discussing findings	Improvement in skills and knowledge during the study. Students stated that EBP might have an influence on increasing the quality of nursing practice.
Balakas [23]	2010	Teaching research and evidence-based practice using a service learning approach	Journal of Nursing Education	USA	Qualitative	Nursing students	A research course from a traditional format to one of evidence appraisal and synthesis, which incorporated service learning and collaborative learning	Research courses taught from an EBP perspective can provide motivation for students to incorporate research into their practice.
Berven [24]	2010	Students collaborate with nurses from a nursing home to get an evidence based practice... Fourth European Nursing Congress	Journal of Clinical Nursing	Norway	Qualitative	Nursing students	Groups of students cooperated with professionals at Løvåsen teaching nursing home in identifying clinical issues that could be feasible to investigate and develop up to date, state-of-art guidelines in relation to model for EBP	Students have developed an understanding that the process of EBP should be utilised in clinical practice.
Blazeck [25]	2011	Building EBP into the foundations of practice	Nurse Educator	USA	Qualitative	Nursing students	Assignment including choosing relevant topic and searching relevant databases	The didactic instruction of the concepts of search and the terminology of search, collaborating with a medical librarian in the teaching and the design of the assignment, the grading rubric for the students, and the quality control visual correction tool for our multiple raters, has led to success
Bloom [26]	2013	Levelling EBP content for undergraduate nursing students	Journal of professional nursing	USA	Qualitative	Nursing students	3 undergraduate research courses designed to prepare the graduate to identify, locate, read and critically appraise evidence at the individual study, systematic review, and clinical practice guideline levels	The foundation achieved by baccalaureate graduates stand them in good stead as they pursue their clinical and academic careers.
Boyd [27]	2015	Using Debates to Teach EBP in Large Online Courses	Journal of Nursing Education	USA	Qualitative	Nursing students	Interactive debates to teach EBP skills in an online graduate course	Students remain highly engaged while practicing critical thinking, teamwork, leadership, delegation, communication skills, and peer evaluation through participation in a series of faculty-facilitated online debates.
Brown [28]	2015	The iPad: tablet technology to support nursing and midwifery student learning: an evaluation in practice	Computers, Informatics, Nursing	USA	Quantitative	Nursing students	Use of iPads	iPads reportedly improved student efficiency and time management, while improving their ability to provide patient education. Students who used iPads for the purpose of formative self-assessment appreciated the immediate feedback and opportunity to develop clinical skills.
Cable-Williams [29]	2014	An educational innovation to foster evidence-informed practice	Journal of Nursing education and Practice	Canada	Qualitative	Nursing students	Threading the concept of evidence-informed practice and relevant best practice guidelines through theory courses including their use as expected elements in clinical placements	The results of research are informing client care and a critical approach to professional practice among nursing students.
Callaghan [30]	2011	Enhancing health students’ understanding of generic research concepts using a web-based video resource	Nurse Education in Practice	England	Qualitative	Physiotherapy students	Innovative video resources	Overall, students perceived the resources as demystifying the topic of research methods through the clarification of definition and application of concepts and making sense of concepts through the analogical videos.
Coyne [31]	2018	A Comprehensive Approach to Undergraduate Nursing Students’ Research Experiences	Journal of Nursing Education	USA	Qualitative	Nursing students	Summer Research Internship (8 weeks during the summer); supporting students in a one-to-one mentorship model with the goal of building a research infrastructure facilitated by researchers and students	The programme leads to practice improvements, knowledge dissemination, and student interest in research and further professional development. It gives students hands-on experience with nursing research that has proven to be beneficial clinically while increasing student interest in research and further nursing education
Crawford [32]	2011	Using problem-based learning in web-based components of nurse education	Nurse Education in Practice	Australia	Qualitative	Nursing students	PBL approaches in online education	Students accessing online nursing subjects would seem to benefit from web-based PBL as it provides flexibility, opportunities for discussion and co-participation, encourages student autonomy, and allows construction of meaning as the problems mirror the real world. PBL also promotes critical thinking and transfer of theory to practice.
Davidson [33]	2016	Teaching EBP using game-based learning: Improving the student experience	Worldviews on evidence-based nursing	Canada	Qualitative	Nursing students	Online EBP course	Students indicated a high satisfaction with the course and student engagement was also maintained throughout the course.
Dawley [34]	2011	Using a pedagogical approach to integrate evidence-based teaching in an undergraduate women’s health course	Worldviews on evidence-based nursing	USA	Qualitative	Nursing students	Pedagogical approach aimed at [1] fostering undergraduate nursing students’ EBP competencies, and [2] identifying gaps in the literature to direct future women’s health research	The assignment was an important teaching and assessment tool for EBP.
Dewar [35]	2012	The EBP course as an opportunity for writing	Nurse Educator	USA	Qualitative	Nursing students	Writing workshops	The workshop approach provides students with a “safe” place to explore their assumptions, learn from peers, and make a leap forward along their personal learning curve as writers.
Doyle [36]	2016	Information Literacy in a Digital Era: Understanding the Impact of Mobile Information for Undergraduate Nursing Students	Book chapter in: Nursing Informatics 2016: eHealth for All: Every Level Collaboration – From Project to Realization	Canada	Qualitative	Nursing students	Use of mobile information resources	Nursing students mainly assessed mobile resources to support clinical learning, and specifically for task-oriented information such as drug medication or patient conditions/diagnoses. Researchers recommend a paradigm shift whereby educators emphasise information literacy in a way that supports evidence-based quality care.
Eales-Reynolds [37]	2012	A study of the development of critical thinking skills	Nurse Education Today	Australia	Qualitative	Nursing students and students from other healthcare disciplines	A novel web 2.0-based tool – the Web Resource Appraisal Process (WRAP)	To ensure that practice developments are based on authoritative evidence, students need to develop critical thinking skills which may be facilitated by tools such as the WRAP.
Elsborg Foss [38]	2014	A model (CMBP) for collaboration between university college and nursing practice to promote research utilization in students’ clinical placements: a pilot study	Nurse Education in Practice	Norway	Quantitative	Nursing students	CMBP (The Collaboration Model of Best Practice)	The CMBP has a potential to be a useful model for teaching RNs’ and students EBP. However, further refinement of the model is needed.
Epstein [39]	2011	Teaching Statistics to Undergraduate Nursing Students: An Integrative Review to Inform our Pedagogy	International Journal of Nursing Education Scholarship	USA	Qualitative	Nursing students	Learning strategies: Schematic links between statistics and everyday nursing practice. Technological Strategies: use of data analysis software (Excel, SPSS etc.) + use of the Internet. Group learning activities: Small group/ workshop activities. Support: student, faculty, −and laboratory support	It was found that there is limited-to-no evidence concerning the pedagogy of statistics.
Erichsen [40]	2018	Kunnskapsbasert praksis i sykepleierutdanningen	Sykepleien Forskning nr. 12,016	Norway	Qualitative	Nursing students	Description of learning-activities including all steps in teaching and conducting EBP	Systematic training in EBP in cooperation with the practice field can have a positive impact on students’ learning. More international and Norwegian research with different study designs is necessary to increase the knowledge.
Florin [41]	2012	Educational support for research utilization and capability beliefsregarding evidence-based practice skills: a national survey of senior nursing students	Journal of AdvancedNursing	Sweden	Quantitative	Nursing students	Educational support for research utilisation and capability beliefs regarding EBP skills	Students reported high capability beliefs regarding evidence-based practice skills, but large differences were found between universities for: stating a searchable question, seeking out relevant knowledge and critically appraising and compiling best knowledge.
Friberg [42]	2013	Changing Essay Writing in Undergraduate Nursing Education Through Action Research: A Swedish Example	Nursing Education Perspectives	Sweden	Qualitative	Nursing students	Workshops and literature review	Action research was found to be a relevant procedure for changing ways of working with literature-based, bachelor degree essays.
Gray [43]	2010	Research odyssey: The evolution of a research partnership between baccalaureatenursing students and practicing nurses	Nurse education Today	USA	Quantitative	Nursing students	A research partnership between baccalaureate nursing students and nurses in two acute care hospitals	The research partnership project facilitated student learning and an appreciation of the research process.
Hande [44]	2017	Leveling Evidence-based Practice Across the Nursing Curriculum	The Journal for Nurse Practitioners - JNP	USA	Qualitative	Nursing students	The article describes evolving EBP competencies related to BSN, MSN, and DSN level.BSN level:Team-based learning, seminars, small group activities, identification of clinical problems, literature search, appraisal of literature, evidence-based project addressing a selected clinical problem for the purposes of improving clinical outcomes	Seamless transition for the development of EBP competencies for nurses at each level of education requires thought, strategically placed objectives and learning activities to be woven into the curriculum and courses. Collaboration among faculty from each educational level must occur. Teaching-learning methods must be appropriate and engaging at each level. Teaching-learning methods must challenge the student to apply and produce scholarly work for dissemination
Henoch [45]	2014	Nursing students’ experiences of involvement in clinical research: an exploratory study	Nurse Education in Practice	Sweden	Quantitative	Nursing students	Students involved as data collectors in a research project	Participation as data collectors in research has the potential to increase interest in nursing research among students.
Hickman [46]	2014	EVITEACH: A study exploring ways to optimise the uptake of EBP to undergraduate nurses	Nurse Education in Practice	Australia	Mixed method	Nursing students	EVITACH to explore strategies to increase undergraduate nursing student’s engagement with EBP and to enhance their knowledge utilisation and translation capabilities	There is little robust evidence to guide the most effective way to build knowledge utilisation and translational skills. Effectively engaging undergraduate nursing students in knowledge translation and utilisation subjects could have immediate and long term benefits for nursing as a profession and patient outcomes.
Jakubec [47]	2013	Students Connecting Critical Appraisal to EPB: A Teaching-Learning Activity for Research Literacy	Journal of Nursing Education	Canada	Qualitative	Nursing students	The Research in Practice Challenge including identifying research problems in practice, searching the literature, and critically evaluating evidence	Students value how the activity highlighted the relevance of research literacy for their practice.
Jalali-Nia [48]	2011	Effect of evidence-based education on Iranian nursing students’ knowledge and attitude	Nursing and Health Sciences	Iran	Quantitative	Nursing students	Evidence-based approach incl. The principles of EBP and PICO. The intervention and the control groups, respectively, were taught through an evidence-based and traditional approach	Significant difference between the average scores for attitude of the groups. No statistical significant difference between the average scores of knowledge.
Janke [49]	2012	Promoting information literacy through collaborative service learning in an undergraduate research course	Nurse Education Today	Canada	Qualitative	Nursing students	Service learning project where students worked in groups, and under the guidance of a nursing instructor and librarian, to answer a question posed by practice-based partners	Evaluation of the project indicated that although the project was challenging and labour intensive students felt they learned important skills for their future practice.
Jelsness-Jørgensen [50]	2014	Does a 3-week critical research appraisal course affect how students perceive their appraisal skills and the relevance of research for clinical practice? A repeated cross-sectional survey	Nurse Education Today	Norway	Quantitative	Nursing students and students from other healthcare disciplines	A 3-week critical research appraisal course	Teaching students’ practical critical appraisal skills improved their view of the relevance of research for patients, future work as well as their own critical appraisal skills.
Johnson [51]	2010	Research and EBP: using a blended approach to teaching and learning in undergraduate nurse education	Nurse Education in Practice	England	Qualitative	Nursing students	A discussion of one module team’s experience of working in a Higher Education Institution within the UK, teaching research and EBP to year two undergraduate nursing and midwifery students	The use of a blended approach to teaching and learning can be beneficial to the nurse educator in a variety of ways if careful consideration is given to the use of technology, the learning styles of the student and access to technology.
Jones [52]	2011	Teaching critical appraisal skills for nursing research	Nurse Education in Practice	Australia	Quantitative	Nursing students	An innovative and quality driven subject to improve critical appraisal and critical thinking skills	Students from both campuses showed considerable improvements in knowledge and confidence in the interpretation and analysis of research findings, in all areas after having completed the subject (assessment).
Keiffer [53]	2018	Engaging Nursing Students: Integrating Evidence-Based Inquiry, Informatics, and Clinical Practice	Nursing Education Perspectives	USA	Qualitative	Nursing students	Workshop format engages the students with technology and digital tools to promote active learning; enhance student collaboration and participation. Varying teaching modalities are employed to engage students and reinforce learning. The students are using the Melnyk et al. model as a framework; ask a clinical question using the PICOT criteria, develop strategies to search, appraise research, use evidence to inform clinical decision-making, design a change, and disseminate the evidence	Well-designed curricula require imagination, creativity, and team effort between theory and clinical faculty. Designing projects applicable to the clinical site provides an avenue for students to engage in EBP while demonstrating the achievement of course learning outcomes.
Kiekkas [54]	2015	Nursing students’ attitudes toward statistics: Effect of a biostatistics course and association with examination performance	Nurse Education Today	Greece	Quantitative	Nursing students	Biostatistics course	Students’ attitudes toward statistics can be improved through appropriate biostatistics courses, while positive attitudes contribute to higher course achievements and possibly to improved statistical skills in later professional life.
Kyriakoulis [10]	2016	Educational strategies for teaching EBP to undergraduate health students: systematic review	Journal of Educational Evaluation for Health Professions	USA	Quantitative	Nursing students and students from other healthcare disciplines	Lectures, tutorials, workshops, conferences, journal clubs, and online sessions or combination of these	Multifaceted approach may be best suited when teaching EBM to health students.
Leach [55]	2016	The impact of research education on student nurse attitude, kill and uptake of evidence	Journal of Clinical Nursing	Australia	Quantitative	Nursing students	Research education programme delivered as two eight-week courses in the third year of education	Research education may have a significant effect on nursing students’ research skills and use of EBP, and minimise barriers to EBP post-education.
Lewis [56]	2016	Diminishing Effect Sizes with Repeated Exposure to EBP Training in Entry-Level Health Professional Students: A Longitudinal Study	Physiotherapy Canada	Canada	Quantitative	Physiotherapy students and students from other healthcare disciplines	Two sequential EBP courses. 1. EBP course was aimed at developing foundational knowledge of and skills in the five steps in EBP. 2. EBP course designed to teach students to apply the steps	Knowledge and relevance changed most meaningfully (i.e., showed the largest effect size) for participants with minimal prior exposure to training. Changes in participants’ confidence and attitudes may require a longer timeframe and repeated training exposure.
Liou [57]	2013	Innovative strategies for teaching nursing research in Taiwan	Nursing Research	Taiwan	Quantitative	Nursing students	Innovative Teaching Strategies for a research course including teamwork, laboratory sessions on how to search for published research articles, experiments and mini research projects (experimental group).Didactic lecture, textbook readings, and research article critique (control group)	This study confirmed that using innovative teaching strategies in nursing research courses enhances student interest and enthusiasm about EBP.
Laaksonen [58]	2013	Journal club as a method for nurses and nursing students’ collaborative learning: a descriptive study	Health Science Journal	Finland	Quantitative	Nursing students	A six-phased journal clubmodel	Journal clubs support competences and discussion required for producing evidence-based care and can be recommended as learning methods for nurses’ and nursing students’ collaborative learning.
Malik [59]	2017	Using pedagogical approaches to influence evidence-based practice integration - processes and recommendations: findings from a grounded theory study	Journal of Advanced Nursing (JAN)	Australia	Qualitative	Nurse academics (regarding nursing students)	Various pedagogical approaches to influence evidence-based practice education; lectures, tutorials, laboratory work, online activities, videos, scenarios, and assignments. Emphasising information literacy and critical appraisal skills. Some use flipped classroom approach, problem-based learning, virtual simulated environment, and inquiry-based learning to facilitate students’ learning	Academics attempted to contextualise EBP by engaging students with activities aiming to link evidence to practice and with the EBP practice. Engaging students with the EBP process in practice context is imperative to increase their EBP competence. Some key challenges (limited time, insufficient resources, heavy workload, students’ disengagement, and limited awareness of effective teaching methods) require the adoption of appropriate strategies to ensure future nurses are well prepared in the paradigm of evidence-based practice
Mattila [60]	2014	Journal club intervention in promoting evidence-based nursing: Perceptions of nursing students	Nurse Education in Practice	Finland	Quantitative	Nursing students	Journal clubs	Students were not able to utilise the studies to the same extent as they learn from them. Age, work experience and participation in research and development activities were connected to learning.
McCurry [61]	2010	Teaching undergraduate nursing research: a comparison of traditional and innovative approaches for success with millennial learners	Journal of Nursing Education	USA	Mixed method	Nursing students	Innovative assignments that included interactive learning, group work, and practical applications	Students’ positive responses to the innovative learning strategies evaluated in this study support the nursing profession’s need to continue to develop activities that engage millennial students and enable them to clearly articulate the value of the research practice link vital to evidence-based nursing practice.
Milner [62]	2017	The PICO Game: An Innovative Strategy for Teaching Step 1 in Evidence-Based Practice	Worldviews on Evidence-Based Nursing	USA	Qualitative	Nursing students	Game	Games build and strengthen skills to frame practice questions in a searchable format (PICO). The method for teaching how to build PICO questions is the same regardless of participant education level or years of practice
Moch [63]	2010	Part II. Empowering grassroots EBP: a curricular model to foster undergraduate student-enabled practice change	Journal of professional nursing	USA	Qualitative	Nursing students	The “Student-Enabled Practice Change Curricular Model”	As the preliminary data reported here suggest, nurse educators have the power to promote practice change by enabling socially meaningful partnerships between students and practicing nurses that could percolate change up from the lowest points in the power hierarchy.
Moch [64]	2010	Part I. Undergraduate nursing EBP education: envisioning the role of students... first of a three-part series	Journal of professional nursing	USA	Qualitative	Nursing students	Various pedagogical strategies targeted towards teaching EBP	The literature reviewed in this article that describes more active roles for students in clinical settings, albeit scant, suggests that allowing students to interact in on-going and meaningful ways with practicing nurses may remove or mitigate barriers to the adoption of EBP among practicing nurses.
Morris [65]	2016	The use of team-based learning in a second year undergraduate pre-registration nursing course on evidence-informed decision making	Nurse Education in Practice	England	Mixed method	Nursing students	Evidence-informed decision making course	Team-based learning was shown to be an effective strategy that preserved the benefits of small group teaching with large student groups.
Morris [66]	2010	Pilot study to test the use of a mobile device in the clinical setting to access evidence-based practice resources	World Views on Evidence-based Nursing	England	Quantitative	Nursing and physiotherapy students	Use of mobile device to access EBP resources in clinical setting	Students reported improvement in knowledge and skills in relation to EBP and appraisal of clinical guidelines. However a low level of utilisation of the mobile device in the clinical setting due to access to the internet and small screens.
Nadelson [67]	2014	Evidence-Based Practice Article Reviews Using CASP Tools: A Method for Teaching EBP	Worldviews on evidence-based nursing	USA	Qualitative	Nursing students	EBP Article Reviews using CASP Tools	Using the CASP Tools help students organise their reviews and learn about valuable resources. In addition, working as a group member helps foster involvement, motivation, and interest in the processes of evaluating evidence effectively.
Nadelson [68]	2014	Online resources: fostering students EBP learning through group critical appraisals	World views on Evidence-based Nursing	USA	Qualitative	Nursing students	Students in dyads or triads reviewed and evaluated one EBP related website	Having students work in groups to critically appraise websites that help promote EBP can enhance collaboration and knowledge about EBP resources.
Niven [69]	2013	Making research real: Embedding a longitudinal study in a taught research course for undergraduate nursing students	Nurse Education Today	USA	Qualitative	Nursing students	To facilitate students learning research theory and methodology by conducting a “real-life” research study in a local retirement community	We knew we had succeeded in our efforts to change student perceptions about learning research when we read a comment from one student who had completed the revised research course.
O’Neil [70]	2016	A new model in teaching undergraduate research: A collaborative approach and learning cooperatives (CALC)	Nurse Education in Practice	USA	Qualitative	Nursing students	A quality improvement study using the CALC Model	Universities and hospital administrators, nurses, and students benefit from working together and learning from each other.
Odell [71]	2011	Teaching EBP: The Bachelor of Science in Nursing Essentials at Work at the Bedside	Journal of professional nursing	USA	Qualitative	Nursing students	A group project for students that involved collaboration with the health science reference librarian and nurse managers in the clinical agencies	The learning experience is a shared partnership between the clinical agency, the faculty, and the health science librarian to assist senior nursing students in the last semester of their baccalaureate degree programme to synthesise and use the knowledge, skills, and attitudes that promote patient safety and optimal outcomes
Oja [72]	2011	Using problem-based learning (PBL) in the clinical setting to improve nursing students’ critical thinking: an evidence review	Journal of Nursing Education	USA	Qualitative	Nursing students	PBL	The studies reviewed indicate a positive relationship between PBL and improved critical thinking in nursing students.
Pennington [73]	2010	EBP partnerships: building bridges between education and practice	Nursing Management	USA	Qualitative	Nursing students	Teaming nursing students with staff nurses working on EBP projects	Students were able to learn how evidence is utilised in the practice settings.
Phelps [74]	2015	Introducing Information Literacy Competency Standards for Nursing	Nurse educator	USA	Qualitative	Nursing students	Information Literacy Competency Standards for Higher Education (ILCSHE)	Nursing librarians are the Information Literacy experts who help to integrate these skills into nursing education
Phillips [75]	2014	Creative classroom strategies for teaching nursing research	Nurse Educator	USA	Qualitative	Nursing students	Kaleidoscopes for discussion of perspectives, crossword puzzles to reinforce terminology, scavenger hunt to relate concepts to the real world, cookie experiment to have an overview of the research process and paradigms, individual reaction time, and a music activity to reinforce elements of design and sampling	Student feedback was positive. These strategies help faculty communicate important concepts of nursing research in a way that is meaningful and fun.
Pierce [76]	2016	The e-Poster Conference: An Online Nursing Research Course Learning Activity	Journal of Nursing Education	USA	Qualitative	Nursing students	e-poster conference	From all accounts, the conference was rated as positive, providing nursing students with opportunities to (a) view studies and projects from a wider nursing science audience, (b) foster the development of important evaluation and communication skills, and (c) be exposed to evidence that could be translated into their practice.
Putnam [77]	2011	Conquering EBP using an embedded librarian and online search tool	Journal of Nursing	USA	Qualitative	Nursing students	Embedded librarians + online search tools to assist students in the development and mastery of effective search techniques	Embedded librarians and online search tools are useful to students as they develop information literacy skills related to searching for and screening information. Using these strategies for formative and summative assignments allows students to develop additional information literacy skills needed to integrate, analyse, apply, and present information.
Raines [78]	2016	A collaborative strategy to bring evidence into practice	Worldviews on evidence-based nursing	USA	Qualitative	Nursing students	A teaching strategy which combines the clinical experience of nurses with nursing students’ evolving skills in reading, critiquing, and analysing research-based literature	The teaching strategy presents a win-win situation in which students become engaged with clinical nurses in a unit-based project.
Raurell-Torreda [79]	2015	Simulation-based learning as a tactic for teaching EBP	Worldviews on evidence-based nursing	Spain	Qualitative	Nursing students	Simulation-based learning (SBL) modules covering nursing competencies	The simulation helped to educate nursing students in applying EBP.
Reicherter [80]	2013	Creating disseminator champions for EBP in health professions education: An educational case report	Nurse Education Today	USA	Qualitative	Nursing and physiotherapy students	A model for developing EBP practitioners: Phase 1. Preparing students how to read, analyse and discuss levels of evidence. Phase 2. Focus on developing dissemination skills by requiring students to complete a clinical case report project. Phase 3. Review outcomes of the project and phase 4. Provide mechanisms of future plans	Increased student participation, Clinical instructors and faculty scholarship, and dissemination of EBP. Additional educational benefits derived from this project included, 1) broader participation of clinical settings, 2) requests by additional clinics to participate for purposes of developing EBP and scholarly presentation skills of clinicians, and 3) increased opportunity for academic faculty to continue engagement in contemporary clinical practice.
Revaitis [81]	2013	FaceTime: a virtual pathway between research and practice	Nurse Educator	USA	Qualitative	Nursing students	FaceTime videoconference	FaceTime videoconferencing provides numerous benefits for students and provides a virtual connection to link the classroom with the practice world.
Roberts [82]	2011	Finding and using evidence in academic assignments: The bane of student life	Nurse Education in Practice	England	Quantitative	Nursing students	Specific sessions on literature searching skills which were delivered early on in the programme	The findings indicate that students value specific teaching sessions (taught by members of library staff) delivered at the beginning of the programme but it seems that more work is required by educators in order to help students to associate literature searching skills with nursing practice.
Rodriguez [83]	2012	Action Research as a Strategy for Teaching an Undergraduate Research Course	Journal of Nursing Education	USA	Qualitative	Nursing students	Teaching of Action Research instead of teaching traditional research course methods	The students learned how to identify a research problem and move through the steps of the research process using action research.
Rolloff [84]	2010	A constructivist model for teaching EBP	Nursing Education Perspectives	USA	Qualitative	Nursing students	Constructivist Model with suggestions of teaching EBP principles during all bachelor years	The constructivist theory for learning may provide a framework for a redesigned baccalaureate curriculum, one that supports EBP throughout a nursing student’s education.
Ruskjer [85]	2010	A real-world experience to engage students in EBP	Journal of Nursing Education	USA	Qualitative	Nursing students	1. Practicing nurses submit clinical questions, 2. Students attend seminar incl. EBP review process + source of evidence, 3. Students select clinical question, appraise systematic reviews and other literature, 4. Faculty consult students incl. Introduction to PICO, 5. Students write abstracts and make power point and poster	Using evidence to answer burning questions straight from the clinical settings is an effective way to engage students and staff nurses in EBP.
Ruzafa-Martinez [86]	2016	Effectiveness of an EBP course on the EBP competence of undergraduate nursing students: A quasi-experimental study	Nurse Education Today	Spain	Quantitative	Nursing students	A 15-week course designed to teach EBP competence	Undergraduate nursing students experience positive changes in EBP competence, knowledge, skills, and attitude as the result of a 15-week educational intervention on EBP.
Schams [87]	2010	Clinical Post-conference Pedagogy: Exploring EBP With Millennial-Inspired ‘Building Blocks’	Creative nursing	USA	Qualitative	Nursing students	Innovative teaching strategy consisting of learning units whereby students come to post-conference sessions prepared to share EBP information associated with upcoming laboratory concepts, discover relationships among laboratory concepts and current nursing practice, and associate personal clinical experiences with the practice environment	Students demonstrated more confidence in questioning current practice, researching EBP literature, and working in groups. The Building Blocks teaching strategy provided an innovative way to engage students during post-conferences to connect practice concepts to real-life experiences, and promoted the use of EBP in guiding practice decisions.
Schreiner [88]	2015	How undergraduate students can contribute to EBP	Nursing Management	Canada	Qualitative	Nursing students	Partnership between university and hospital working together on EBP- projects	Students involved in the pilot programme expanded their research horizon and learned to conduct literature reviews, utilize search engines, and categorize articles. Being involved in clinical research can be an asset to undergraduate students for future practice and education.
Scott [89]	2011	A collaborative teaching strategy for enhancing learning of evidence-based clinical decision-making	Journal of Allied Health	USA	Qualitative	Occupational -and physiotherapy students	Partnership between university and hospital	The approach increased student motivation and greatly enhanced the learning experience.
Scurlock-Evans [90]	2017	To embed or not to embed? A longitudinal study exploring the impact of curriculum design on the evidence-based practice profiles of UK pre-registration nursing students	Nurse Education today	UK	Quantitative	Nursing students	This study compared the impact of embedding EBP throughout the curriculum, with modular-based teaching, on pre-registration nursing students’ EBP profiles.	Taking a modular or embedded approach to EBP may have little impact on students’ final EBP profiles
Sin [91]	2017	Teaching evidence based practice to undergraduate nursing students	Journal of Professional Nursing	USA	Qualitative	Nursing students	A group project designed in a Nursing Research Methods course. The project was based on a hypothetical clinical scenario and students were not asked to implement the best intervention	Nursing faculty is responsible for preparing students to be ready for EBP implementation. Creative and enjoyable teaching strategies are some ways to enhance students’ knowledge and competency of EBP implementation in practice
Smith-Stoner [92]	2011	Developing new writers: answering the call for student manuscripts	Dimensions of Critical Care Nursing	USA	Qualitative	Nursing students	Students participated in a critical-care rotation and were enrolled in an introductory research class	During a recent critical-care nursing rotation, nursing students learned about EBP through identifying a policy that needed revision or creation. By integrating clinical issues into an introduction to research and issues and trends, the students were able to answer a call for student abstracts.
Smith-Strøm [93]	2012	Culture crash regarding nursing students’ experience of implementation of EBP in clinical practice	Nordic Journal of Nursing Research	Norway	Qualitative	Nursing students	12 day course in EBP steps + collaboration with clinical practice to apply the steps of EBP	The students were able to implement EBP according to the goals of the syllabus, but encountered a clinical setting that was insufficiently prepared, both structurally and in terms of knowledge, to mentor them regarding EBP.
Stombaugh [94]	2013	Using lesson study to integrate information literacy throughout the curriculum	Nurse Educator, 2013, Canada	Canada	Qualitative	Nursing students	Lesson study	The lesson study method is an ideal way to implement a scaffolding approach of teaching information literacy skills towards EBP outcomes.
Strickland [95]	2012	The use of podcasts to enhance research-teaching linkages in undergraduate nursing students	Nurse Education in Practice	Scotland	Quantitative	Nursing students and students from other healthcare disciplines	Blended learning approach. Students were given access to a series of 5 “guest speaker” podcasts made up of presentations and interviews with research experts	Podcasting offers nurse educators the ability to embed additional content from researchers or clinicians to help students make links between their theoretical learning and practice.
Sukkarieh-Haraty [96]	2017	Integrating Evidence-Based Practice into a Lebanese Nursing Baccalaureate Program: Challenges and Successes	International Journal of Nursing Education Scholarship	Libanon	Qualitative	Nursing students	Two courses at two different levels. Students used PICO clinical question, observed a selected clinical skill and compared their observations to hospital protocol and against the latest evidence-based practice guidelines. At the second course students proposed changes in practice with scholarly literature.	An overall experience of integrating EBP project into the curriculum is fruitful for students, clinical agencies, and faculty. Students gain real-life skills needed for EBP.
Whalen [97]	2015	Teaching Systematic Searching in a Baccalaureate Nursing Research Course	World views on Evidence-based Nursing	USA	Quantitative	Nursing students	Implementing systematic worksheets and research logs on students’ EBP projects	Students who did not use systematic search worksheets and research logs scored significantly lower on evidence summaries than students using systematic search worksheets and research logs.
Wonder [98]	2015	Active learning strategies to teach undergraduate nursing statistics: Connecting class and clinical to prepare students for EBP	Worldviews on evidence-based nursing	USA	Quantitative	Nursing students	Active learning strategy:Students are presented with a case scenario via PowerPoint to start the active learning experience.Small groups/ each person collects data individually (chocolate chip cookies, exercise). Methodological and statistical discussions	The active learning exercises and assignments had a positive impact on students’ academic and clinical development. Students reported that by beginning with simple exercises that allowed them to visualise and physically touch data, it enabled them to progress to more abstract and complex applications.
Yu [99]	2013	Improvement in critical thinking dispositions of undergraduate nursing students through problem-based learning: a crossover-experimental study	Journal of Nursing Education	China	Quantitative	Nursing students	One group receiving problem-based learning (PBL) and the other group receiving lecture-based learning (LBL) as a control	PBL is an effective method to improve the quality of medical teaching and the abilities of nursing students, as well as a means to improve implementation of knowledge, ability, and quality, but it also presents an effective means to improve critical thinking dispositions in nursing students in China.
Zhang [100]	2012	Assisting undergraduate nursing students to learn evidence-based practice through self-directed learning and workshop strategies during clinical practicum	Nurse Education Today	China	Quantitative	Nursing students	A pilot learning programme including a self-directed learning process for EBP basics and a workshop for critical appraisal of literature	Significant improvement in students’ perception of EBP knowledge, attitudes, beliefs, and behaviour. Students found the programme helpful in promoting their analytical and problem-solving abilities.

### Key methods for teaching EBP, the Sicily Statement’s five steps of teaching and conducting EBP and context

Table 3 presents the key methods for teaching EBP, the Sicily Statement’s five steps of teaching and conducting EBP, and the context. Seven key methods for teaching EBP were identified: Thirty-two studies described “Research courses and workshops”. “Collaboration with clinical practice” was identified 14 times followed by “IT technology” (*n* = 8), “Assignments” (*n* = 5), “Participation in research projects” (*n* = 5), “Journal clubs” (*n* = 2), and “Embedded librarians” (*n* = 2). In addition, 13 studies described various theories of teaching and learning. These are not elaborated on as the theme is not considered within the objective of this scoping review.

**Table 3:** Key methods for teaching undergraduate healthcare students EBP, the Sicily Statement’s five steps of teaching and conducting EBP and context

Source (first author, year)	Key methods for teaching undergraduate healthcare students EBP	The Sicily Statement’s five steps in teaching and conducting EBP					Context
		1. Ask a clinical question	2. Collect the most relevant evidence	3. Critically appraise the evidence	4. Integrate the evidence with one’s clinical expertise, patient preferences, and values to make practice decision	5. Evaluate change or outcome	
Balakas, 2010 [23]	Research courses and workshops	Students learned how to use their clinical PICO question…	..as a guide for conducting literature searches	Students were guided in the use of rapid appraisal guidelines for quantitative and qualitative research. Written critical appraisals were completed to further develop students’ critiquing skills	Each student group presented their PICO questions, evidence synthesis, reference list, and recommendations to the community programme managers	Students learned to evaluate a body of evidence	Classroom + clinical practice
Bloom, 2013 [26]	Research courses and workshops		Nursing Science I: The process of reviewing the literature is explored, and the final project for the course is a literature search designed to identify the most current evidence available for a given topic	Nursing Science II: The emphasis of the course is on critical appraisal of a primary research report	Nursing Science III: Students use evidence-based models to systematically practice decision-making skills related to a clinical question of interest to them		Classroom
Boyd, 2015 [27]	Research courses and workshops						Classroom
Cable-Williams, 2014 [29]	Research courses and workshops						Classroom + clinical practice
Davidson, 2016 [33]	Research courses and workshops	Students learn to develop PICO clinical questions…	…searches for external evidence to answer focused clinical questions…	…participates in the critical appraisal of published research studies…	…to determine their strength and applicability to clinical practice…	…and disseminates best practices supported by evidence to improve quality of care and patient outcomes	Classroom
Dewar, 2012 [35]	Research courses and workshops	Four 3-h writing workshops including how to develop a clinical question…		…and identify relevant information from published researchstudies			Classroom
Friberg, 2013 [42]	Research courses and workshops		Students had a close collaboration with librarians with ten different workshops focusing on different aspects of literature retrieval	Students used knowledge-based analysis of both quantitative and qualitative results…	…and best evidence for a specific nursing action and transformed results and new knowledge into practice		Classroom
Jakubec, 2013 [47]	Research courses and workshops	Students wrote their appraisal of evidence in an existing policy or guideline…	…met with a health reference librarian to conduct a systematic search of the literature on the topic…	…provided a critical review of existing evidence with the policy or guideline and reviewed any updated or more recent evidence…	…and wrote a summary of their recommended policy changes for practice		Classroom
Jalali-Nia, 2011 [48]	Research courses and workshops	The evidence-based approach, learning activities for each group included developing a clinical question using the PICO…	…searching for evidence…	…reading and critiquing nursing research…	… and discussing articles, synthesising the evidence, and developing a summary of findings		Classroom
Janke, 2012 [49]	Research courses and workshops	Students had to clarify the research question…	…designing a literature search strategy and complete the search…	…select the articles and record important data from the articles…	…and submit the paper/results to the clinical partners		Classroom
Jelsness-Jørgensen, 2015 [50]	Research courses and workshops		Week 1: Lectures in databases and literature search	Week 1: Introduction to Critical Appraisal Skill Tools. Week 2: Group work and seminars focusing on critical appraisal of qualitative papers. Week 3: Group work and seminars focusing on critical appraisal of quantitative papers			Classroom
Jones, 2011 [52]	Research courses and workshops			The assessment tasks were designed to enable students to conduct and report a critique of a published paper	The third and fourth assessment tasks were designed to enable students to apply the skills they had learnt in the subject		Classroom
Kiekkas, 2015 [54]	Research courses and workshops						Classroom
Kyriakoulis, 2016 [10]	Research courses and workshops	Interventions covered different steps of the EBP domains: Research question…	…sources of evidence…2 studies focused on the searching databases skill	…evidence appraisal…	…and implementation into practice…		Classroom
Leach, 2016 [55]	Research courses and workshops	Identification and development of research question from practice	Construction and execution of search strategies to retrieve relevant primary research articles	Critical appraisal of the literature	Summary, presentation and dissemination of evidence in different formats		Classroom + clinical practice
Lewis, 2016 [56]	Research courses and workshops	The EBP1 course aimed to develop foundation knowledge and skills in EBP, with emphasis on three of the five EBP steps outlined in the Sicily Statement incl. Frame a research question…	…to access and search library databases and other resources and to reflect on the processes associated with this approach.	The EBP2 course had additional training inAppraising methodological bias…	…as well as teaching students how to apply each of the five EBP steps		Classroom
Liou, 2013 [57]	Research courses and workshops	Mini research project with introduction how to formulate a research problem…	…conduct literature searches…	…read and select articles…	…and an oral and poster presentation of findings		Classroom
Morris, 2016 [65]	Research courses and workshops						Classroom
Phillips, 2014 [75]	Research courses and workshops						Classroom
Pierce, 2016 [76]	Research courses and workshops	During the e-poster conference students develop a research question…	…appraise data collection…	…critique published literature…	…and write about how to begin a change to organisational visitation policy based on the research evidence from the poster conference		Classroom
Rodriguez, 2012 [83]	Research courses and workshops		Students conducted a research project which included a literature review…		…presented their results and designed a scientific poster with their results		Classroom
Whalen, 2015 [97]	Research courses and workshops	The worksheet included mainly step 1–3 of EBP. Asking a clinical question using PICO…	…searching the literature…	…and critically appraising the literature found			Classroom
Zhang, 2012 [100]	Research courses and workshops		Students independently conducted online and library searches to find information	Students were asked to read an assigned article and critique it to the best of their ability	Students created presentation slides and shared an in-depth critique of one aspect of the specified research article		Classroom + clinical practice
Milner, 2017 [62]	Research courses and workshops	Students learn to build and frame practice questions by gaming					Classroom
Sukkarieh-Haraty, 2017 [96]	Research courses and workshops	Students learned how to use a clinical PICO question	…and collected scholarly literature	Compared their observations to hospital protocol against the latest evidence-based practice guidelines	Students proposed changes in practice with scholarly literature		Classroom + clinical practice
Erichsen, 2018 [40]	Research courses and workshops	Ask a clinical question	Collect relevant literature/articles	Critically appraise the articles	Students present their work in different ways; e.g. implementation-plan, poster	The results were evaluated	Classroom + clinical practice
Scurlock-Evans, 2017 [90]	Research courses and workshops		Students were taught what EBP is, how it links with research methodology and process and ethics (in year 2)	Students were taught how to assess quality of literature/evidence (in year 1)	Students undertook an independent research project in their final year (3 year)		Classroom
Keiffer, 2018 [53]	Research courses and workshops	Students ask a PICOT (population, intervention, control, outcomes, time) question	Develop strategies to search – and search	Appraise research	Design a change and disseminate the evidence by making recommendations for best practice		Classroom
Sin, 2017 [91]	Research courses and workshops	Faculty have framed questions/students develop a question using PICO later in their nursing school	Acquiring evidence by selecting evidence-based resources through literature in collaboration with a librarian	Students state the rationale for their intervention choice incorporating the appraisal learned in the class	Students are asked to identify at least three EBP implementation strategies based on their literature review using at least two references		Classroom
Coyne, 2018 [31]	Research courses and workshops	Students learned how to ask research questions and how to lean on one another for help and guidance	Students helped the faculty member in her research project to collect relevant literature	…including helping with initial review of the literature	Students did a formal podium presentation regarding their summer experiences. The programme led to changes at the health system and led to initiation of research studies		Classroom + clinical practice
Hande, 2017 [44]	Research courses and workshops	Students identify the potential clinical questions as they become aware of current generalist nursing care problems	Students are guided through the sequence of steps to review research	Students critically appraise the scholarly information	Students are guided through the sequence of steps to develop an EBP implementation plan	Students make a presentation of an evidence-based project addressing a selected clinical problem for the purposes of improving clinical outcomes: Population/patient, problem, intervention, comparison, outcome, time question, recommendations for evidence-based practice change	Classroom + clinical practice
Malik G, 2017 [59]	Research courses and workshops	Asking clinical questions	Finding relevant evidence (sometimes workshops delivered by the library staff)	Appraising the evidence	Applying evidence into clinical practice (theoretically)		Classroom
Berven, 2010 [24]	Collaboration with clinical practice						Clinical practice
Elsborg Foss, 2014 [38]	Collaboration with clinical practice		Students were taught in computer-based literature search	Students read, appraised, and discussed the articles that were chosen	Students presented the findings from the literature search about ‘best practice’ and the recommendations for changes…	…and second-year students observed to what extent the decisions about changes were followed	Classroom
Gray, 2010 [43]	Collaboration with clinical practice	In the introductory nursing research course prior to the research partnership, all nursing students are required to complete an evidence-based research project including the five steps					Classroom + clinical practice
Moch, 2010 [64]	Collaboration with clinical practice						Classroom
Moch, 2010 [63]	Collaboration with clinical practice		In discussion groups students found four articles related to the topic…	…and students and staff, along with faculty, read and discussed each of the articles in four discussion sessions			Classroom
Odell & Barta, 2011 [71]	Collaboration with clinical practice		Assignment outcomes related to step 2 and 3: Collaborate in the collection of evidence and participate in the process of appraisal, of evidence				Clinical practice
O’Neal, 2016 [70]	Collaboration with clinical practice	Students wrote a related PICOT question…	…conducted a review of the literature	…followed guidelines to critically appraise articles	…identified application to practice	…developed recommendation for the future	Clinical practice
Pennington, 2010 [73]	Collaboration with clinical practice	Students wrote up the formalised research proposal	Students performed literature searches and…	…were instruments in collection and analysis of the pre-implementation survey data	The partnerships offered students + staff an opportunity to experience how make best practice decisions using a systematic EBP process		Classroom + clinical practice
Raines, 2016 [78]	Collaboration with clinical practice		Students searched relevant evidence and…	…reviewed the literature found and appraised the quality of the evidence found			Classroom + clinical practice
Reicherter, 2013 [80]	Collaboration with clinical practice	Students learn to develop an evidence-based question…	…search for and retrieve relevant journal articles…	…analyse the results…	…student teams create and present a case report to classmates and outline potential clinical decisions using the evidence		Classroom + clinical practice
Schams, 2012 [87]	Collaboration with clinical practice	Students were encouraged to write a clinical question using PICOT.	The group was divided into teams who shared the responsibilities for searching and reporting EBP information that supported or refuted current practice. As a team students discussed relationships among laboratory concepts, current practice, and EBP information found in literature. By using post-conference time immediately following clinical practice experiences, students could associate their personal experiences in practice with the EBP information.				Classroom + clinical practice
Scott, 2011 [89]	Collaboration with clinical practice	Students learned to write PICOT questions…	…and search the literature	Students learned appraisal and met with therapists to validate direction of search…	…and relevance of evidence to practice		Classroom + clinical practice
Smith-Stoner, 2011 [92]	Collaboration with clinical practice		Students performed literature searches…		…and presented editing policy to clinical staff		Clinical practice
Smith-Strøm, 2012 [93]	Collaboration with clinical practice	The 12 –day course trained the students in the four steps of EBP: Formulating a question…	…searching for evidence…	…critically appraising the evidence…	…and applying the evidence		Clinical practice
Brown, 2015 [28]	IT Technology		The iPad provided point-of-care access to clinical guidelines and resources…		…enabling students to implement an evidence-based approach to decision making and problem solving		Classroom
Callaghan, 2011 [30]	IT technology		Staff revealed two key research processes as being vital to students’ understanding of research and subsequent critical appraisal, these being searching for…	…and evaluating literature			Classroom
Doyle, 2016 [36]	IT technology		Mobile software is a positive information tool for information literacy…		…and for informing clinical decisions		Clinical practice
Eales-Reynolds, 2012 [37]	IT technology			Students indicated that the WRAP improved their critical appraisal skills…	…and questioning of the research evidence basis for practice		Classroom
Morris, 2010 [66]	IT technology	The guideline appraisal activity helped students formulated a searchable question	The guideline appraisal activity helped students retrieve evidence	The guideline appraisal activity helped students critically appraise the evidence	The guideline appraisal activity helped students apply the evidence to practice		Clinical practice
Nadelson, 2014 [68]	IT technology			Critical group appraisals of EBP websites relevant for clinicians			Classroom
Revaitis, 2013 [81]	IT technology				Through FaceTime videoconference students benefit from interacting with research teams and are able to discuss how research findings are applied to practice		Classroom
Strickland, 2012 [95]	IT technology						Classroom
Blazeck, 2011 [25]	Assignments		The main purpose of the assignment is accessing research-based evidence relevant to an identified clinical problem				Classroom
Dawley, 2011 [34]	Assignments	Students were to generate relevant clinical questions that evolved from their clinical experiences…	…and were asked to conduct a literature search to identify two research articles that began to answer their questions				Classroom
McCurry, 2010 [61]	Assignments		Students completed a database search and met with the course faculty to refine electronic searches…	…critically examined the literature…	…and submitted abstracts and prepared an oral presentation and poster of the chosen articles		Classroom
Nadelson, 2014 [67]	Assignments			Students receive an article to be reviewed, read and critically appraise using the CASP tool			Classroom
Roberts, 2011 [82]	Assignments		Students learned to search the literature using a variety of mechanisms				Classroom
Andre, 2016 [22]	Participation in research projects			Increased understanding of the importance of critical thinking	Increased understanding of the importance of implementation of research in daily practice	Increased understanding of the importance of evaluation of clinical practice through the use of EBP	Classroom + clinical practice
Henoch, 2014 [45]	Participation in research projects		Students collected data				Classroom + clinical practice
Niven, 2013 [69]	Participation in research projects		Students collected both qualitative and quantitative data using questionnaires				Classroom + clinical practice
Ruskjer, 2010 [85]	Participation in research projects	Faculty guide the team in constructing the question in PICO	Librarian provides guidance in the computer laboratory, as students gain hands-on experience conducting an online literature search	The team critically appraises systematic reviews and practice guidelines, and individual students appraise relevant research articles	Faculty assists the team in looking at the evidence and discusses any recommended changes in practice		Classroom
Schreiner, 2015 [88]	Participation in research projects		Students initiated the project by conducting a literature review for EBP articles related to heart failure education	Articles were chosen by their relevance to the enhancement of staff education for heart failure patients			Clinical practice
Laaksonen, 2013 [58]	Journal clubs		Students searched for scientific knowledge to answer a clinical question of the journal club…	…evaluated the articles and other relevant material…	…and prepared short written papers based on the knowledge they had collected and evaluated		Classroom
Mattila, 2013 [60]	Journal clubs		Students prepared for the journal club by acquiring data with the help of an information specialist		After presenting the article, participants discussed how the results could be used in nursing care and what type of solution or new perspective had been gained. Students generated the discussion and gave their opinion of the both oral and written		Classroom
Phelps, 2015 [74]	Embedded librarians		The ILCSN will help students gather…	…analyse…	…and use information		Classroom
Putnam, 2011 [77]	Embedded librarians		The embedded librarian assisted students in developing appropriate search techniques	The summative EBP paper developed the review of literature, including integrating, analysing…	…applying, and presenting information		Classroom
Aglen, 2016 [21]	Theories of teaching – and learning	The pedagogical strategies presented invite the learner to become an active participant in the learning activity, e.g. assessing research, conducting a research project and assessing patients’ requirements for healthcare. This means that they are encouraged to use discretion to solve ill-structured problems related to the steps of EBP, the research process and their own clinical practice. Another strategy to enhance students’ interest and make the learning tasks relevant is to link the learning task to real clinical situations					Classroom
Crawford, 2011 [32]	Theories of teaching – and learning			PBL enhances critical thinking…	..and transfer of theory to practice		Classroom + clinical practice
Epstein, 2011 [39]	Theories of teaching – and learning						Classroom
Florin, 2012 [41]	Theories of teaching – and learning	Highest correlation coefficients between students’ experience of support for research utilisation and EBP skills in formulating questions to search for research-based knowledge (step 1) and critically appraising and compiling best knowledge (step 3) on campus.					Classroom + clinical practice
Hickman, 2014 [46]	Theories of teaching – and learning						Classroom
Johnson, 2010 [51]	Theories of teaching – and learning	Students develop their own research proposal, which includes defining a research question…	…searching the literature…		…and formulate appropriate methods		Classroom
Oja, 2011 [72]	Theories of teaching – and learning			All studies except one in the review found significant effects of PBL on critical thinking skills			Clinical practice
Raurell-Torredà, 2015 [79]	Theories of teaching – and learning						Classroom + clinical practice
Rolloff, 2010 [84]	Theories of teaching – and learning	Students will develop information literacy skills…	…explore systematic review databases for evidence related to laboratory experiences and introduce other information literacy sources	…critique websites, research articles and clinical experiences from an EBP perspective for health information	…incorporate EBP into patient care plans and develop a research proposal based on evidence gaps identified in practice	…and evaluate clinical policies and procedures from an EBP perspective and discuss change process	Classroom
Ruzafa-Martinez, 2016 [86]	Theories of teaching – and learning	Students should identify a nursing problem in patients cared for during clinical training and formulate a clinical PICO question...	…identify clinical practice guidelines, systematic reviews and/or original articles…	…critically appraise search results…	…describe recommendations on the clinical question and identify the level of evidence and grade of recommendation…	…and present the results of the final exercise in a poster to the seminar group, giving reasons for implementation of the search results	Classroom
Stombaugh, 2013 [94]	Theories of teaching – and learning	Sophomore-level: Students generated a PICO question…Students copied the process of the librarian describing an example of a PICO question, creation of a search term and conduction of a search in CINAHL Junior level: Students searched databases other than CINAHL Senior level: Students created PICO related to practice experience, individually searched databases and retrieved “best practice” evidence					Classroom
Wonder, 2015 [98]	Theories of teaching – and learning			Students critically appraised analysis methods and findings in the context of quality and safety improvement…	…and identified implications for nursing and the inter-professional team		Classroom
Yu, 2013 [99]	Theories of teaching – and learning						Classroom

In Table 3 the vast majority of the studies (*n* = 69) referred to one or more of the Sicily Statement’s five steps of teaching and conducting EBP. Eleven studies referred to all five steps. Thirty-one studies referred to three or four steps, while 17 studies referred to two of the steps, and ten studies described one step. Twelve studies had no description of any of the steps.

The steps most often referred to were step two, three, and four. Step two, “Collect the most relevant evidence”, was mentioned in 58 studies. Step three, “Critically appraise the evidence”, was referred to in 55 studies, while step four, “Integrate the evidence with one’s clinical expertise, patient preferences, and values to make practice decision”, was mentioned in 51 studies. Step one, “Ask a clinical question”, and step five, “Evaluate change or outcome” was referred to in 36 and 14 studies, respectively. Seven out of the eleven studies referring to all of the Sicily Statement’s five steps were identified under the key methods “Research courses and workshops” and “Collaboration with clinical practice”.

The context in which the studies were conducted was primarily classroom settings (*n* = 52). Twenty studies described context as a combination of classroom and clinical practice, and nine studies were conducted in clinical practice.

Out of the 68 studies which included the seven key methods, 24 out of 32 “Research courses and workshops” were conducted in classrooms, while “Collaboration with clinical practice” was conducted in a combination of classroom and clinical practice (*n* = 6), clinical practice (*n* = 5), or classrooms (*n* = 3). “IT technology” was used in classrooms (*n* = 6) or clinical practice (*n* = 2). “Assignments” were conducted in classroom settings only (*n* = 5), while “Participation in research projects” took place in a combination of classroom and clinical practice (*n* = 3), classroom (*n* = 1) or clinical practice (*n* = 1). “Embedded librarians” (*n* = 2) and “Journal clubs” (*n* = 2) both took place in classroom settings.

## Discussion

This study provides an overview of existing EBP teaching methods including The Sicily Statement’s steps of teaching and conducting EBP with respect to undergraduate healthcare students both in classrooms and in clinical practice.

It is beyond the scope of this review to interpret all the findings of the included studies. The findings discussed below are the key methods most frequently identified in the thematic analysis: “Research courses and workshops” and “Collaboration with clinical practice”, as well as the key methods most positively referred to in main findings of the studies: “IT technology”, “Embedded librarians”, and “Journal clubs”. Despite the scarce use of the last three methods (“IT technology”, “Embedded librarians”, “Journal clubs”) these can however provide ideas for how to teach EBP in the future. Furthermore, the scoping review provides useful information as to which of the Sicily Statement’s five steps of teaching and conducting EBP are taught in the various methods and whether one particular method is more useful and applicable than others in a particular learning setting, depending on the context and the learning outcomes. Lecturers, senior lecturers and others who teach EBP at undergraduate healthcare educational institutions can benefit from this information and gain inspiration and ideas for teaching EBP. We are aware that other studies have addressed issues such as teachers’ competencies required for teaching EBP, which we do consider important in order to standardise and improve education in EPB. Interestingly, a study has identified specific sets of EBP core competencies for teachers, which are classified within the 5-step model of EBP [101]. However, since our primary focus was on methods for teaching EBP to undergraduate healthcare students the aspect of teacher’s competencies has not been further investigated.

Overall, the first 4 steps of the Sicily Statement could more easily be identified. However the last and fifth step proved to be more difficult to identify which often is the case. Furthermore, our results tend to point to the fact that the fifth step is often more theoretically linked at the undergraduate level and that a more specified implemtation and evaluation of the EBP process takes place at a more advanced level.

### Research courses and workshops

Research courses and workshops were the most frequently used methods for teaching EBP. The frequent use of this method is in agreement with the systematic review by Kyriakoulis et al., where eight of the 20 methods for teaching EBP were research courses, workshops or similar sessions [10] and Young et al., where three out of five methods used for teaching evidence-based healthcare (EBHC) were workshops [2]. The majority of the studies concerning “Research courses and workshops”, referred to three or more of the Sicily Statement’s five steps of teaching and conducting EBP [10, 23, 26, 31, 33, 40, 42, 44, 47–49, 53, 55–57, 59, 76, 90, 91, 96, 97, 100]. Despite the fact that the fifth step was included in some studies, it was not clear what was covered by the evaluation process, and additionally, if it was part of the students’ assignment work alone or if there was a link to clinical practice. The majority of the research courses and workshops were conducted in classrooms. According to Young et al., EBHC courses can improve appraisal skills in nurses, occupational therapists and physiotherapists, among others [2]. However, further assessments and analyses of the courses and workshops found in this scoping review must be made to ensure that the content and outcomes are applicable in similar contexts.

### Collaboration with clinical practice

In this scoping review, the key method for teaching EBP, “Collaboration with clinical practice” was identified 14 times in the thematic analysis. In comparison, “collaboration with clinical practice” is only mentioned once as a method for teaching EBP in the review by Young et al. [2] and not mentioned in the systematic review by Kyriakoulis et al. [10]. The results of this scoping review suggest that collaboration with clinical practice is a frequently used method for teaching EBP with respect to undergraduate healthcare students. The rare use of this method in the review by Kyriakoulis et al. and the review by Young et al. might be explained by the type of participants included in these reviews. Only two studies included undergraduate students in the disciplines of nursing, physiotherapy or occupational therapy: in the review by Young et al. and the review by Kyriakoulis [2, 10]. Along this line, a review on teaching EBM to medical students found weak and inconsistent results from a limited number of studies on the effect of clinically integrated methods on knowledge, attitudes, and skills [102]. Collaboration with clinical practice might be more fundamental among undergraduate students in the disciplines of nursing, physiotherapy or occupational therapy, compared to undergraduate students in medicine primarily included in the other reviews [2, 10].

Half of the studies identified in relation to “Collaboration with clinical practice”, referred to four or five of the Sicily Statement’s steps [38, 43, 70, 73, 80, 87, 89, 93]. In two of the studies, the steps were taught directly in clinical practice as part of the students’ clinical education [70, 93], and unlike the research courses and workshops methods, the last step of evaluation of change or outcome is carried out either partly or entirely in a clinical context. The main findings of all eight studies indicate that collaboration with clinical practice is an effective way of teaching EBP, both with the combination of classrooms and clinical practice settings and in clinical practice settings alone. Despite being a recommended strategy in the literature, a recent literature review points to the fact that EBP teaching strategies including clinical activities in nursing students seems less prioritised [12].

### IT technology

The key method, “IT technology”, described tools, such as mobile devices, video resources and websites, among others, used in classrooms or clinical practice for seeking information in relation to EBP. In clinical practice, mobile devices were used to seek information regarding EBP search strategies, critical appraisal of clinical guidelines [66], and specific task-oriented information in relation to clinical practice [36]. Despite the reported improvement in knowledge and skills in relation to EBP and appraisal of clinical guidelines, the use of mobile devices was reported as low [66]. In classrooms, the use of IT technology as a method for teaching EBP was reported as mainly positive. Today, most students have access to IT equipment and this technology could be integrated in classrooms and clinical practice to seek information regarding EBP. In the review by Kyriakoulis et al. the results support our findings; that IT technology can be an effective method for teaching EBP with respect to undergraduate healthcare students [10].

### Journal clubs

“Journal Clubs” as a method for teaching EBP was only described in two studies in this scoping review. However, the findings indicated that the method improved students’ skills in reading articles and understanding evidence-based nursing [60], and promoted competenceis needed to deliver evidence-based care [58]. The studies included two and three steps, respectively, proposed by the Sicily Statement [3]. Additional steps may advantageously be incorporated into future journal clubs to ensure the quality of healthcare. Young et al. referred to four reviews describing a positive effect of journal clubs as a method for teaching EBP [2]. However, none of the reviews included Professional Bachelor Degree healthcare students. Further studies must be conducted to assess the effectiveness of journal clubs as a method for teaching EBP in Professional Bachelor Degree healthcare courses and to study the opportunity of incorporating all of the Sicily Statement’s five steps for teaching and conducting EBP.

### Embedded librarians

In two studies librarians were introduced to teach students information literacy [74, 77]. Librarians are experts in this field and are able to teach students the skills essential to EBP [74]. Librarians can support students in establishing and managing effective search techniques and help with reviewing and critiquing the information found. Thus, students develop information literacy skills as required in practice [77]. Research librarians who are embedded as part of a research course for teaching undergraduate healthcare students EBP may be an effective way of ensuring a complete introduction to the Sicily Statement’s five steps of teaching and conducting EBP. The first three steps can be taught in classrooms, followed by practical exercises in the last two steps in clinical practice.

### Implications for practice

The majority of the key methods found for teaching EBP were “Research courses and workshops” and “Collaboration with clinical practice”, whereas “Journal Clubs” and “Embedded librarians” were identified only twice. The frequent use of research courses and workshops as methods for teaching EBP may stem from a tradition of classroom lectures and is a relatively manageable way to teach EBP. The findings from this scoping study however suggest that other methods for teaching EBP with respect to undergraduate students exist. Journal clubs could be incorporated as a supplement to classroom lectures or as part of collaboration with clinical practice. Librarians may advantageously be introduced in research courses and workshops. The librarians can help students gain control of EBP definitions and concepts, and master search techniques before entering clinical practice. The effect of journal clubs and embedded librarians on students’ EBP competencies should however be investigated further before being incorporated into Professional Bachelor’s Degree curricula.

EBP education based on the Sicily Statement’s five steps, demanded both internationally and nationally, implies an introduction to all of the Sicily Statement’s five steps of teaching and conducting EBP at undergraduate level [5, 6, 9]. At present, the majority of the methods found for teaching EBP only include 2–4 steps. In line with these results, a recent review found that the majority of evaluated EBP educational interventions are focused on a single step (step 3, critically appraising evidence) of the five steps of teaching and conducting EBP [103]. This research adds to the statement that an effort must be made to incorporate all five steps of the Sicily Statement in an educational and cooperative way in order to ensure that undergraduate healthcare students are qualified to work in an evidence-based manner.

We did not review available assessment methods for evaluation of EBP education interventions or programmes in this study. The additional importance of this field and the apparent lack of valid evaluation methods have been extensively highlighted in other studies and needs to be taken into account when applying methods for teaching EPB [104, 105].

### Strengths and limitations

The scoping review presents an updated overview of existing methods for teaching EBP with respect to undergraduate healthcare students, including study specific recommendations for teaching methods to be used in future curricula. It follows recommended guidelines for a priori design requirements and transparent reporting [13, 16].

Limitations are however found in relation to the search strategy. Our search did not include literature published before 2010 and due to time constraints, a limited number of databases were searched, which entails the risk that not all relevant literature was identified. Furthermore, the search terms used identified primarily undergraduate nursing students, despite a thorough search for all undergraduate students in the disciplines of nursing, physiotherapy, occupational therapy, radiography, and biomedical laboratory science. Other search terms might have captured these health disciplines to a greater extent. An alternative explanation for the large occurrence of studies regarding nursing students could be that there is a greater publishing tradition in this field. However, the methods for teaching EBP can to a certain extent, be introduced to undergraduate students in the other health disciplines.

## Conclusion

Consistent with our objective, this scoping review has provided an extensive overview of literature describing methods for teaching EBP regarding undergraduate healthcare students. The two key methods most often identified were “Research courses and workshops” and “Collaboration with clinical practice”. Despite the first method often being used in this scoping review, as well as in other reviews, fewer of the Sicily Statement’s five steps of teaching and conducting EBP were referred to, and if the fifth step of evaluation of change or outcome was included, the description of content was often unclear.

On the contrary, “Collaboration with clinical practice”, the second most used teaching method, more often referred to four or five steps, making this method an effective approach for teaching EBP while ensuring incorporation of several of the steps. Unlike the Research courses and workshop methods, the last step of evaluation is carried out partly or entirely in a clinical context.

Overall, our results tend to show that the evaluation step is often theoretically linked at the undergraduate level. Despite the small number of studies describing ‘Journal clubs’ and ‘Embedded librarians’, these teaching methods could advantageously be incorporated in the classroom context and could ensure a complete introduction to all five steps.

On the basis of our findings, we argue that future research should focus on identifying methods for teaching EBP that incorporate as many of the Sicily Statement’s five steps of teaching and conducting EBP as possible. Journal clubs and embedded librarians could be further looked into as methods to support the more established methods for teaching EBP across all undergraduate healthcare disciplines.

## Data Availability

The data used and analysed during the current scoping review are available from the corresponding author on reasonable request.

## References

[1] Dizon JM, Grimmer-Somers K, Kumar S. Effectiveness of the tailored Evidence Based Practice training program for Filipino physical therapists: a randomized controlled trial. BMC Med Educ. 2014;14. 10.1186/1472-6920-14-147. 10.1186/1472-6920-14-147 PMC4131475 25034409

[2] Young T, Rohwer A, Volmink J, Clarke M (2014). What are the effects of teaching evidence-based health care (EBHC)? Overview of systematic reviews. PLoS One.

[3] Dawes M, Summerskill W, Glasziou P, Cartabellotta A, Martin J, Hopayian K (2005). Sicily statement on evidence-based practice. BMC Med Educ.

[4] Burns HK, Foley SM (2005). Building a foundation for an evidence-based approach to practice: teaching basic concepts to undergraduate freshman students. J Prof Nurs.

[5] Daly M, DeAngelis T (2017). Teaching evidence-based practice across curricula—an overview of a professional development course for occupational therapy educators. Occup Ther Health Care.

[6] Greenhalg T, Howick J, Maskrey N (2014). Evidence-based medicine: a movement in crisis?. BMJ.

[7] Styrelsen for Videregående Uddannelser, Uddannelses- og Forskningsministeriet (2015). Direction for the work of single profession development groups.

[8] Ministry of Higher Education and Science (2016). The Danish higher education system.

[9] Kristensen H, Nøhr-Jensen L, Stokholm G, Berg P, Borg-Hansen K, Pedersen K, et al. Evidence-based practice - Health educations University College Lillebaelt - Curiculum revision. 2016;1:2–10.

[10] Kyriakoulis K, Patelarou A, Laliotis A, Wan AC, Matalliotakis M, Tsiou C (2016). Educational strategies for teaching evidence-based practice to undergraduate health students: systematic review. J Educ Eval Health Prof.

[11] Ghaffari R, Shapoori S, Binazir MB, Heidari F, Behshid M (2018). Effectiveness of teaching evidence-based nursing to undergraduate nursing students in Iran: a systematic review. Res Dev Med Educ.

[12] Horntvedt MT, Nordsteien A, Fermann T, Severinsson E (2018). Strategies for teaching evidence-based practice in nursing education: a thematic literature review. BMC Med Educ.

[13] Munn Z, Peters MD, Stern C, Tufanaru C, McArthur A, Aromataris E (2018). Systematic review or scoping review? Guidance for authors when choosing between a systematic or scoping review approach. BMC Med Res Methodol.

[14] Merriam-Webster (2017). Classroom.

[15] Segen’s Medical Dictionary (2011). Clinical practice.

[16] The Joanna Briggs Institute (2015). The Joanna Briggs Institute reviewer’s manual: 2015 edition / supplement.

[17] Joanna Briggs Institute (2019). JBI reviewer’s manual.

[18] Communiqué of the Conference of European Ministers Responsible for Higher Education (2009). The Bologna Process 2020 - The European higher education area in the new decade.

[19] Paniagua H (2002). Planning research: methods and ethics. Pract Nurs.

[20] Cronin P, Ryan F, Coughlan M (2008). Undertaking a literature review: a step-by-step approach. Br J Nurs.

[21] Aglen B (2016). Pedagogical strategies to teach bachelor students evidence-based practice: a systematic review. Nurse Educ Today.

[22] Andre B, Aune AG, Braend JA (2016). Embedding evidence-based practice among nursing undergraduates: results from a pilot study. Nurse Educ Pract.

[23] Balakas K, Sparks L (2010). Teaching research and evidence-based practice using a service-learning approach. J Nurs Educ.

[24] Berven L (2010). Students collaborate with nurses from a nursing home to get an evidence based practice...Fourth European Nursing Congress. J Clin Nurs.

[25] Blazeck A, Klem ML, Miller TH (2011). Building evidence-based practice into the foundations of practice. Nurse Educ.

[26] Bloom KC, Olinzock BJ, Radjenovic D, Trice LB (2013). Leveling EBP content for undergraduate nursing students. J Prof Nurs.

[27] Boyd MR, Baliko B, Polyakova-Norwood V (2015). Using debates to teach evidence-based practice in large online courses. J Nurs Educ.

[28] Brown J, McCrorie P (2015). The iPad: tablet technology to support nursing and midwifery student learning: an evaluation in practice. Comput Inform Nurs.

[29] Cable-Williams B, Rush J, Mowry A, Macleod A, Gilmer C, Graham C (2014). An educational innovation to foster evidence-informed practice. J Nurs Educ.

[30] Callaghan L, Lea SJ, Mutton L, Whittlesea E (2011). Enhancing health students’ understanding of generic research concepts using a web-based video resource. Nurse Educ Pract.

[31] Coyne BM, Kennedy C, Self A, Bullock L (2018). A comprehensive approach to undergraduate nursing students’ research experiences. J Nurs Educ.

[32] Crawford TR (2011). Using problem-based learning in web-based components of nurse education. Nurse Educ Pract.

[33] Davidson SJ, Candy L (2016). Teaching EBP using game-based learning: improving the student experience. Worldviews Evid Based Nurs.

[34] Dawley K, Bloch JR, Suplee PD, McKeever A, Scherzer G (2011). Using a pedagogical approach to integrate evidence-based teaching in an undergraduate women’s health course. Worldviews Evid Based Nurs.

[35] Dewar SR (2012). The evidence-based practice course as an opportunity for writing. Nurse Educ.

[36] Doyle GJ, Furlong KE, Secco L (2016). Information literacy in a digital era: understanding the impact of mobile information for undergraduate nursing students. Stud Health Technol Inform.

[37] Eales-Reynolds LJ, Gillham D, Grech C, Clarke C, Cornell J (2012). A study of the development of critical thinking skills using an innovative web 2.0 tool. Nurse Educ Today.

[38] Elsborg Foss J, Kvigne K, Wilde Larsson B, Athlin E (2014). A model (CMBP) for collaboration between university college and nursing practice to promote research utilization in students’ clinical placements: a pilot study. Nurse Educ Pract.

[39] Epstein I, Santa Mina EE, Gaudet J, Singh MD, Gula T. Teaching statistics to undergraduate nursing students: an integrative review to inform our pedagogy. Int J Nurs Educ Scholarsh. 2011;8(1). 10.2202/1548-923X.2234.

[40] Erichsen T, Røkholt G, Utne I (2018). Kunnskapsbasert praksis i sykepleierutdanningen. Sykepleien Forskning.

[41] Florin J, Ehrenberg A, Wallin L, Gustavsson P (2012). Educational support for research utilization and capability beliefs regarding evidence-based practice skills: a national survey of senior nursing students. J Adv Nurs.

[42] Friberg F, Lyckhage ED (2013). Changing essay writing in undergraduate nursing education through action research: a Swedish example. Nurs Educ Perspect.

[43] Gray MT (2010). Research odyssey: the evolution of a research partnership between baccalaureate nursing students and practicing nurses. Nurse Educ Today.

[44] Hande K, Ty Williams C, Robbins HM, Kennedy BB, Christenbery T (2017). Leveling evidence-based practice across the nursing curriculum. J Nurse Pract.

[45] Henoch I, Jakobsson Ung E, Ozanne A, Falk H, Falk K, Kenne Sarenmalm E (2014). Nursing students’ experiences of involvement in clinical research: an exploratory study. Nurse Educ Pract.

[46] Hickman LD, Kelly H, Phillips JL (2014). EVITEACH: a study exploring ways to optimise the uptake of evidence-based practice to undergraduate nurses. Nurse Educ Pract.

[47] Jakubec SL, Astle BJ (2013). Students connecting critical appraisal to evidence-based practice: a teaching-learning activity for research literacy. J Nurs Educ.

[48] Jalali-Nia SF, Salsali M, Dehghan-Nayeri N, Ebadi A (2011). Effect of evidence-based education on Iranian nursing students’ knowledge and attitude. Nurs Health Sci.

[49] Janke R, Pesut B, Erbacker L (2012). Promoting information literacy through collaborative service learning in an undergraduate research course. Nurse Educ Today.

[50] Jelsness-Jørgensen L (2015). Does a 3-week critical research appraisal course affect how students perceive their appraisal skills and the relevance of research for clinical practice? A repeated cross-sectional survey. Nurse Educ Today.

[51] Johnson N, List-Ivankovic J, Eboh WO, Ireland J, Adams D, Mowatt E (2010). Research and evidence based practice: using a blended approach to teaching and learning in undergraduate nurse education. Nurse Educ Pract.

[52] Jones SC, Crookes PA, Johnson KM (2011). Teaching critical appraisal skills for nursing research. Nurse Educ Pract.

[53] Keiffer MR. Engaging Nursing Students: Integrating Evidence-Based Inquiry, Informatics, and Clinical Practice. Nurs Educ Perspect. 2018;39(4):247-249. 10.1097/01.NEP.0000000000000235 29215391

[54] Kiekkas P, Panagiotarou A, Malja A, Tahirai D, Zykai R, Bakalis N (2015). Nursing students’ attitudes toward statistics: effect of a biostatistics course and association with examination performance. Nurse Educ Today.

[55] Leach MJ, Hofmeyer A, Bobridge A (2016). The impact of research education on student nurse attitude, skill and uptake of evidence-based practice: a descriptive longitudinal survey. J Clin Nurs.

[56] Lewis LK, Wong SC, Wiles LK, McEvoy MP (2016). Diminishing effect sizes with repeated exposure to evidence-based practice training in entry-level health professional students: a longitudinal study. Physiother Can.

[57] Liou SR, Cheng CY, Tsai HM, Chang CH (2013). Innovative strategies for teaching nursing research in Taiwan. Nurs Res.

[58] Laaksonen C, Paltta H, von Schantz M, Ylönen M, Soini T (2013). Journal club as a method for nurses and nursing students’ collaborative learning: a descriptive study. Health Sci J.

[59] Malik G, McKenna L, Griffiths D (2017). Using pedagogical approaches to influence evidence-based practice integration - processes and recommendations: findings from a grounded theory study. J Adv Nurs.

[60] Mattila L, Rekola L, Koponen L, Eriksson E (2013). Journal club intervention in promoting evidence-based nursing: perceptions of nursing students. Nurse Educ Pract.

[61] McCurry MK, Martins DC (2010). Teaching undergraduate nursing research: a comparison of traditional and innovative approaches for success with millennial learners. J Nurs Educ.

[62] Milner KA, Cosme S (2017). The PICO game: an innovative strategy for teaching step 1 in evidence-based practice. Worldviews Evid-Based Nurs.

[63] Moch SD, Cronje RJ (2010). Part II. Empowering grassroots evidence-based practice: a curricular model to foster undergraduate student-enabled practice change. J Prof Nurs.

[64] Moch SD, Cronje RJ, Branson J (2010). Part 1. Undergraduate nursing evidence-based practice education: envisioning the role of students. J Prof Nurs.

[65] Morris J (2016). The use of team-based learning in a second year undergraduate pre-registration nursing course on evidence-informed decision making. Nurse Educ Pract.

[66] Morris J, Maynard V (2010). Pilot study to test the use of a mobile device in the clinical setting to access evidence-based practice resources. Worldviews Evid-Based Nurs.

[67] Nadelson S, Nadelson LS (2014). Evidence-based practice article reviews using CASP tools: a method for teaching EBP. Worldviews Evid Based Nurs.

[68] Nadelson SG (2014). Online resources: fostering students evidence-based practice learning through group critical appraisals. Worldviews Evid-Based Nurs.

[69] Niven E, Roy DE, Schaefer BA, Gasquoine SE, Ward FA (2013). Making research real: embedding a longitudinal study in a taught research course for undergraduate nursing students. Nurse Educ Today.

[70] O'Neal PV, McClellan LC, Jarosinski JM (2016). A new model in teaching undergraduate research: a collaborative approach and learning cooperatives. Nurse Educ Pract.

[71] Odell E, Barta K (2011). Teaching evidence-based practice: the bachelor of science in nursing essentials at work at the bedside. J Prof Nurs.

[72] Oja KJ (2011). Using problem-based learning in the clinical setting to improve nursing students’ critical thinking: an evidence review. J Nurs Educ.

[73] Pennington K, Moscatel S, Dacar S, Johnson C (2010). EBP partnerships: building bridges between education and practice. Nurs Manag.

[74] Phelps SF, Hyde L, Planchon WJ (2015). Introducing information literacy competency standards for nursing. Nurse Educ.

[75] Phillips RM (2014). Creative classroom strategies for teaching nursing research. Nurse Educ.

[76] Pierce LL (2016). The e-poster conference: an online nursing research course learning activity. J Nurs Educ.

[77] Putnam J, Faltermeier D, Riggs CJ, Pulcher K, Kitts R (2011). Conquering evidence-based practice using an embedded librarian and online search tool. J Nurs Educ.

[78] Raines DA (2016). A collaborative strategy to bring evidence into practice. Worldviews Evid Based Nurs.

[79] Raurell-Torredà M, Romero-Collado À (2015). Simulation-based learning as a tactic for teaching evidence-based practice. Worldviews Evid Based Nurs.

[80] Reicherter EA, Gordes KL, Glickman LB, Hakim EW (2013). Creating disseminator champions for evidence-based practice in health professions education: an educational case report. Nurse Educ Today.

[81] Revaitis M, Egger S (2013). FaceTime: a virtual pathway between research and practice. Nurse Educ.

[82] Roberts D, Ousey K (2011). Finding and using evidence in academic assignments: the bane of student life. Nurse Educ Pract.

[83] Rodriguez R (2012). Action research as a strategy for teaching an undergraduate research course. J Nurs Educ.

[84] Rolloff M (2010). A constructivist model for teaching evidence-based practice. Nurs Educ Perspect.

[85] Ruskjer B (2010). A real-world experience to engage students in evidence-based practice. J Nurs Educ.

[86] Ruzafa-Martinez M, Lopez-Iborra L, Armero Barranco D, Ramos-Morcillo AJ (2016). Effectiveness of an evidence-based practice (EBP) course on the EBP competence of undergraduate nursing students: a quasi-experimental study. Nurse Educ Today.

[87] Schams KA, Kuennen JK (2012). Clinical postconference pedagogy: exploring evidence-based practice with millennial-inspired ‘building blocks’. Creative Nurs.

[88] Schreiner M, Kudrna B, Kenney C (2015). How undergraduate students can contribute to EBP. Nurs Manag.

[89] Scott PJ, Altenburger PA, Kean J (2011). A collaborative teaching strategy for enhancing learning of evidence-based clinical decision-making. J Allied Health.

[90] Scurlock-Evans L, Upton P, Rouse J, Upton D (2017). To embed or not to embed? A longitudinal study exploring the impact of curriculum design on the evidence-based practice profiles of UK pre-registration nursing students. Nurse Educ Today.

[91] Sin MK, Bliquez R (2017). Teaching evidence based practice to undergraduate nursing students. J Prof Nurs.

[92] Smith-Stoner M (2011). Developing new writers: answering the call for student manuscripts. Dimens Crit Care Nurs.

[93] Smith-Strøm H, Oterhals K, Rustad EC, Larsen T (2012). Culture crash regarding nursing students’ experience of implementation of EBP in clinical practice. Vard i Norden.

[94] Stombaugh A, Sperstad R, Vanwormer A, Jennings E, Kishel H, Vogh B (2013). Using lesson study to integrate information literacy throughout the curriculum. Nurse Educ.

[95] Strickland K, Gray C, Hill G (2012). The use of podcasts to enhance research-teaching linkages in undergraduate nursing students. Nurse Educ Pract.

[96] Sukkarieh-Haraty O, Hoffart N. Integrating evidence-based practice into a Lebanese nursing baccalaureate program: challenges and successes. Int J Nurs Educ Scholarsh. 2017;14(1). 10.1515/ijnes-2017-0026. 10.1515/ijnes-2017-0026 28976908

[97] Whalen KJ, Zentz SE (2015). Teaching systematic searching in a baccalaureate nursing research course. Worldviews Evid-Based Nurs.

[98] Wonder AH, Otte JL (2015). Active learning strategies to teach undergraduate nursing statistics: connecting class and clinical to prepare students for evidence-based practice. Worldviews Evid Based Nurs.

[99] Yu D, Zhang Y, Xu Y, Wu J, Wang C (2013). Improvement in critical thinking dispositions of undergraduate nursing students through problem-based learning: a crossover-experimental study. J Nurs Educ.

[100] Zhang Q, Zeng T, Chen Y, Li X (2012). Assisting undergraduate nursing students to learn evidence-based practice through self-directed learning and workshop strategies during clinical practicum. Nurse Educ Today.

[101] Albarqouni L, Hoffmann T, Straus S, Olsen NR, Young T, Ilic D (2018). Core competencies in evidence-based practice for health professionals: consensus statement based on a systematic review and Delphi survey. JAMA Netw Open.

[102] Ahmadi S, Baradaran HR, Ahmadi E (2015). Effectiveness of teaching evidence-based medicine to undergraduate medical students: a BEME systematic review. Med Teach.

[103] Albarqouni L, Hoffmann T, Glasziou P (2018). Evidence-based practice educational intervention studies: a systematic review of what is taught and how it is measured. BMC Med Educ.

[104] Tilson JK, Kaplan SL, Harris JL, Hutchinson A, Ilic D, Niederman R (2011). Sicily statement on classification and development of evidence-based practice learning assessment tools. BMC Med Educ.

[105] Shaneyfelt T, Baum KD, Bell D, Feldstein D, Houston TK, Kaatz S (2006). Instruments for evaluating education in evidence-based practice: a systematic review. JAMA.

